# DCA Protects against Oxidation Injury Attributed to Cerebral Ischemia-Reperfusion by Regulating Glycolysis through PDK2-PDH-Nrf2 Axis

**DOI:** 10.1155/2021/5173035

**Published:** 2021-10-19

**Authors:** Xiaoyong Zhao, Shan Li, Yunchang Mo, Ruru Li, Shaoyi Huang, Anqi Zhang, Xuqing Ni, Qinxue Dai, Junlu Wang

**Affiliations:** ^1^Department of Anesthesiology, The First Affiliated Hospital of Wenzhou Medical University, Wenzhou, 325000 Zhejiang Province, China; ^2^Shandong Provincial Medicine and Health Key Laboratory of Clinical Anesthesia, School of Anesthesiology, Weifang Medical University, Weifang 261021, China

## Abstract

Cerebral ischemic stroke (IS) is still a difficult problem to be solved; energy metabolism failure is one of the main factors causing mitochondrion dysfunction and oxidation stress damage within the pathogenesis of cerebral ischemia, which produces considerable reactive oxygen species (ROS) and opens the blood-brain barrier. Dichloroacetic acid (DCA) can inhibit pyruvate dehydrogenase kinase (PDK). Moreover, DCA has been indicated with the capability of increasing mitochondrial pyruvate uptake and promoting oxidation of glucose in the course of glycolysis, thereby improving the activity of pyruvate dehydrogenase (PDH). As a result, pyruvate flow is promoted into the tricarboxylic acid cycle to expedite ATP production. DCA has a protective effect on IS and brain ischemia/reperfusion (I/R) injury, but the specific mechanism remains unclear. This study adopted a transient middle cerebral artery occlusion (MCAO) mouse model for simulating IS and I/R injury in mice. We investigated the mechanism by which DCA regulates glycolysis and protects the oxidative damage induced by I/R injury through the PDK2-PDH-Nrf2 axis. As indicated from the results of this study, DCA may improve glycolysis, reduce oxidative stress and neuronal death, damage the blood-brain barrier, and promote the recovery of oxidative metabolism through inhibiting PDK2 and activating PDH. Additionally, DCA noticeably elevated the neurological score and reduced the infarct volume, brain water content, and necrotic neurons. Moreover, as suggested from the results, DCA elevated the content of Nrf2 as well as HO-1, i.e., the downstream antioxidant proteins pertaining to Nrf2, while decreasing the damage of BBB and the degradation of tight junction proteins. To simulate the condition of hypoxia and ischemia in vitro, HBMEC cells received exposure to transient oxygen and glucose deprivation (OGD). The DCA treatment is capable of reducing the oxidative stress and blood-brain barrier of HBMEC cells after in vitro hypoxia and reperfusion (H/R). Furthermore, this study evidenced that HBMEC cells could exhibit higher susceptibility to H/R-induced oxidative stress after ML385 application, the specific inhibitor of Nrf2. Besides, the protection mediated by DCA disappeared after ML385 application. To sum up, as revealed from the mentioned results, DCA could exert the neuroprotective effect on oxidative stress and blood-brain barrier after brain I/R injury via PDK2-PDH-Nrf2 pathway activation. Accordingly, the PDK2-PDH-Nrf2 pathway may play a key role and provide a new pharmacology target in cerebral IS and I/R protection by DCA.

## 1. Introduction

Stroke refers to a vital cause of death and permanent disability globally [1], of which ischemic stroke (IS) takes up more than 87% of its incidence [2]. The early intervention strategy of IS refers to restoring the blood supply of infarcted and ischemic areas. Nevertheless, reperfusion is likely to further aggravate ischemic brain injury, i.e., cerebral ischemia/reperfusion (I/R) injury [3, 4]. According to related studies and reports, CIRI displays a relationship with energy metabolism disorder [5, 6], oxidative stress [7, 8], Ca^2+^ overload, excitatory neurotransmitters, apoptosis, and necrosis [9]. Energy metabolism disorder can lead to considerable ROS generation, and oxidative stress attributed to ROS displays a close relationship with IS pathogenesis. In CIRI, the excessive production of ROS will cause DNA, proteins, and brain lipids to undergo oxidative damage, thereby causing cell death and neurological dysfunction [10]. Thus, energy metabolizers are taken into account to prevent and treat IS.

During reperfusion after cerebral ischemia, the blood supply will restore glucose and oxygen levels, produce excessive ROS, promote responses of promoting oxidative stress such as leukocyte and proinflammatory neutrophil infiltration and complement and platelet activation, and damage the blood-brain barrier (BBB) [11, 12], which are all components of reperfusion injury. Accordingly, it is necessary to reduce reperfusion injury to promote cell repair and ischemic tissue regeneration.

Dichloroacetic acid (DCA) is a small molecule, which has been used as a therapeutic agent for many genetic mitochondrial diseases [13, 14]. Dichloroacetic acid (DCA) is an inhibitor of pyruvate dehydrogenase kinase (PDK), and PDK2 is the most abundant isoenzyme in the rat brain [15]. DCA can inhibit mitochondrial PDK2 and activate pyruvate dehydrogenase (PDH), which is a gatekeeper enzyme combining anaerobic (glycolysis) with aerobic (Krebs cycle) metabolism [16, 17]. After ischemia and hypoxia, the activity of PDH decreases, pyruvate cannot decarboxylate and will produce lactic acid via glycolysis, and each glucose molecule produces two moles of ATP. However, when PDH is activated, pyruvate can decarboxylate to acetyl-CoA, enter the tricarboxylic acid cycle, and produce up to 36 moles of ATP per glucose molecule within mitochondria [18]. DCA exerts protecting effects on I/R injury [12], cancer [19], and pulmonary hypertension [20]. Nevertheless, it is not clear if DCA exerts a protecting effect on IS and CIRI.

In this study, we found that mitochondrial-related enzymes are inactivated after cerebral ischemia-reperfusion, and then, glycolysis produces considerable ROS. DCA can improve glycolysis by inhibiting PDK2 and activating PDH, so as to activate Nrf2, reduce oxidative stress, and reduce the permeability of the blood-brain barrier. Thus, as suggested from the results of this study, DCA is likely to be a new therapeutic approach in terms of IS and CIRI.

## 2. Materials and Methods

### 2.1. Sigma-Aldrich (St. Louis, Missouri, USA) Offered Materials

Dichloroacetic acid (347795), 2,3,5-triphenyltetrazole chloride (TTC), and ML385 (GC19254) originated from GLPBIO (Montclair, California, the United States of America). Gibco (Grand Island, NY) provided fetal bovine serum (FBS) and trypsin.

### 2.2. Animal and Animal Experiments

An animal and focal cerebral ischemia model and male C57BL/6 mice aged 6 to 8 weeks were used for this study. The animal operations received approval from the Animal Experimentation Ethics Committee (No. WYDW2019-0559). In addition, the humanistic care was carried out according to the animal experiment guidelines of Wenzhou Medical University. The researchers carried out the transient MCAO model of mice by occluding MCA [21]. In terms of sham-operated mice, the isolation was conducted on the right common and external carotid artery, whereas there was no MCA ligation. The mice received the random allocation in 4 cohorts, i.e., sham cohort, MCAO cohort, DCA (100 mg/kg) cohort, and DCA (200 mg/kg) cohort. After 90 min of occlusion, 100 mg/kg and 200 mg/kg DCA were given to the DCA cohort as soon as the plug was released.

### 2.3. Neurological Deficit Assessment

When the 24 h reperfusion was conducted, the neurological deficit received the assessment in accordance with the scoring standards [22, 23]: 0: no neurological deficit; 1: falling to contralateral sides; 2: failing to have spontaneous activities; 3: failing to stretch the contralateral forelimb; 4: circling to paretic sides.

### 2.4. Infarct Volume Assessment

After neurological assessment, as mentioned before [24], mice were euthanized with 2% pentobarbital sodium, and the mice received the decapitation. The brains received the removal to measure infarct volume. Coronal section slices were taken from the whole brain and then received the staining process with 2% TTC under the temperature of 37°C for 20 min. To conduct the investigation, the pictures of slices were captured by using a digital camera, and all images were collected and analyzed with the use of ImageJ (National Institutes of Health, USA). The relative infarct volume rate was obtained, and the edema was corrected. In brief, the calculating process is conducted below: corrected infarct volume ratio = [contralateral hemisphere area − (ischemic hemisphere area − infarct area)/contralateral hemisphere area] × 100%.

### 2.5. Brain Water Level

The mice received the sacrifice 24 h when MCAO was caused. The brains received careful removal. By weighing the ischemic hemisphere, the wet weight received the rapid measurement. By weighing the samples dried under the temperature of 105°C for 24 h, the dry weight received the measurement. The brain water content is expressed as brain water content (%) = (wet weight − dry weight)/wet weight × 100%.

### 2.6. Nissl Staining

The mice were deeply anesthetized when the neurological deficit test was performed. The left ventricle received the perfusion by using 4% paraformaldehyde. When the perfusion was achieved, the brain received the 48 h fixing process, the dehydration, and the embedment in wax. Coronal sections with a thickness of 10 *μ*m were set for Nissl staining. The experiment was carried out according to the instructions of the Nissl staining tool (Solarbio, China). Brain slices received dehydration by using alcohol and were impregnated with xylene and stained with thiophane. Then, the morphological changes of cortical neurons were observed under a microscope. The number of surviving neurons was recorded by neuron count.

### 2.7. TUNEL Assay

*By* complying with the manufacturer's guidelines, the researchers carried out the TUNEL test using the in situ cell death detection tool (Roche) and detected under the fluorescence microscope (DM2500; Leica Microsystems, Germany). With the use of Image-Pro Plus version 6.0 (Media Cybernetics, USA), the researchers without any knowledge regarding the grouping assignment measured TUNEL positive cells with green fluorescence. Results had the expression of labeled cell numbers.

### 2.8. Assessment of BBB Permeability

Based on the measurement of the penetration of Evans blue (Sigma) in brain tissues, the researchers examined BBB permeability [25]. Evans blue (2% saline, 4 mL/kg body weight) was administered intravenously through the tail vein 1 h before measurement. The anesthetized animals were perfused with normal saline before sampling. The respective sample received the weighing and homogenizing processes by using 400 *μ*L PBS, and subsequently, the sample received the precipitation throughout the night by using 50% trichloroacetic acid. The sample underwent 30 min centrifugation at 10,000 rpm to precipitate the brain tissues. EB absorbance received the measurement at 610 nm using one microplate reader (BioTek, Winooski, Vermont). The concentration was then calculated according to the standard curve, with the expression of *μ*g/g brain tissue.

### 2.9. Electron Microscope Study

The brain slices were fixed with 0.1 mol/L methylarsonic acid buffer of 4% glutaraldehyde (pH 7.4). The slices were then immersed in 1% osmium tetroxide in 0.1 M methyl arsenate buffer for 2 h and stained overnight with 1% uranyl acetate aqueous solution. The tissue sections received the dehydration to 100% with ascending series of ethanol and subsequently with acetone and underwent the embedding process within an epoxy resin. The ultrathin sections received the restaining by using lead citrate before the examination under transmission electron microscopy (H7650).

### 2.10. Cell Culture and Treatment

HBMEC (HUM-CELL-0101) cells were purchased from PriCells (Wuhan, China). The cells received the culture under the temperature of 37°C, 5% CO_2_, and 95% humidity supplemented with 1% penicillin/streptomycin solution (P/S, Sclencell), 1% endothelial cell growth supplement (ECGS, Sclencell), and 10% fetal bovine serum (FBS, Sclencell). To simulate ischemia-like conditions in vitro, HBMEC cells received the transfer toward sugar-free medium and the culture within the Tri-GAS (1% O_2_/5% CO_2_/94% N_2_) incubating tool for 4 h. Subsequently, the glucose-free medium received the replacement by using fresh maintenance medium and the recovery based on normoxic conditions for 24 h.

### 2.11. Determination of Cell Viability (Cell-Counting-Kit 8 (CCK-8) Colorimetric Assay)

Cell viability was determined by CCK-8 (Dojindo Molecular Technologies, Inc., Kumamoto, Japan). For HBMEC cells, the cells are inoculated in 96-well plates with a density of 5,000 cells per well. The next day, HBMEC cells were pretreated with different concentrations of DCA, i.e., 2.5 mM, 5 mM, and 10 mM, 6 h before hypoxia, and then exposed to OGD for 4 h, followed by oxygenation for 24 h. Next, the addition of 20 *μ*L CCK-8 was used to each well, and the incubation was achieved under the temperature of 37°C. Lastly, the absorbance at 450 nm received the measurement with a microplate analyzer.

### 2.12. Oxidative Stress Detection

The brain tissue and HBMEC cells were homogenized and centrifuged with 12000 mg × g for 15 min. The supernatant was collected for spectrophotometric study. The BCA assay kit was adopted for determining the protein concentration. The contents of superoxide dismutase (SOD) and malondialdehyde (MDA) in brain tissue and HBMEC cells were detected by using the appropriate kit (Beyotime Biotechnology, China) and in accordance with the producer's guideline.

### 2.13. Determination of Intracellular Reactive Oxygen Species

To determine the production of intracellular reactive oxygen species, the 2′,7′-dichlorofluorescein diacetate (DCFH-DA) assay was used to measure ROS according to the manufacturer's instructions (Solarbio, Beijing, China). In the presence of ROS, DCFH reacts with ROS to form DCF, a fluorescent product. Intracellular detection of ROS in different groups was achieved by incubating cells with 10 *μ*mol/L DCFH-DA at 37°C in darkness for 30 min. The fluorescence of DCFH-DA is inspired at 488 nm, and the emission is collected at 525 nm. The fluorescence microscope (Olympus, Japan) is used to detect the fluorescence value.

### 2.14. Western Blotting Assay

Overall proteins from the ischemic side cerebral cortex and HBMEC cells received the collection and the fractionation by using SDS-PAGE gels [23]. Subsequently, the protein received the incubation by using primary antibodies against PDK2 (1 : 1000, Abcam, USA), PDH (1 : 1000, Abcam, USA), ZO-1 (1 : 1000, Abcam, USA), occludin (1 : 1000, Abcam, USA), Nrf2 (1 : 500, Proteintech, Chicago, USA), HO-1 (1 : 500, Proteintech, Chicago, USA), and Tubulin (1 : 10000, BaoDragon, Hefei, China). The BCA test kit (P0012; Beyotime Biotechnology) was used to measure protein concentrations. After the denaturation, the same amount of protein was separated by SDS-PAGE and transferred to PVDF membranes (Millipore, Billerica, MA). After the membrane transfer, the membrane was sealed with 5% skim milk at ambient temperatures for 2 h. Next, the membrane received the incubation under the temperature of 4°C with primary antibody and then with appropriate secondary antibody at ambient temperatures for 1 h. Image Lab Software (Bio-Rad Laboratories Inc., Berkeley, CA) was used to detect the protein bands.

### 2.15. Statistical Analysis

All data, in addition to the neurologic score, had the expression of the mean ± standard deviation (S.D.) and received the comparison by ANOVA and then with Tukey's multiple-comparison examination. The neurologic scores had the expression of the median (range) and received the comparison with a nonparametric method (Kruskal-Wallis test) as well as the Mann–Whitney *U* statistic with Bonferroni correction. The researchers employed GraphPad Prism 7.0 (GraphPad, San Diego, CA, USA) to achieve the statistical investigation. A value of *P* < 0.05 was statistically significant.

## 3. Results

### 3.1. DCA Protects Mice from Cerebral Ischemia-Reperfusion Injury

**Figure 1:**
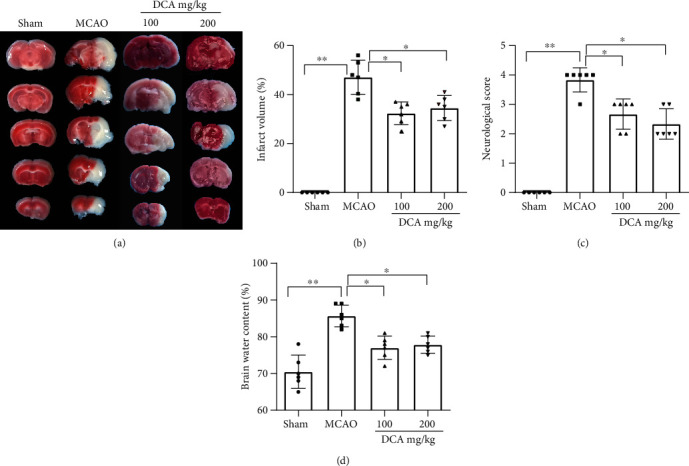
DCA protects mice from brain I/R damage. (a, b) Representative photograph and relative infarct size of mouse coronal slices stained with TTC at 24 h by MCAO (*n* = 6). (c) The effect of DCA on neurological deficit score (*n* = 6). (d) Effect of DCA on brain water content (*n* = 6). In conclusion, the mentioned findings strongly support the protective role of DCA in brain I/R injury in mice. ^∗^*P* < 0.05; ^∗∗^*P* < 0.01. Comparison between cohorts is marked in the figure.

According to Figure 1, to study the potential effect exerted by DCA in CIRI, neurological score, cerebral infarct area rate, and brain edema content were examined 24 h when MCAO was caused. In contrast to sham operation, the MCAO cohort had an increase in cerebral infarct size and cerebral edema and a decrease in neurological scores. The cerebral infarction area and neurological score were significantly improved in the DCA cohort (Figures 1(a)–1(c)). In addition, the DCA treatment significantly improved cerebral edema (Figure 1(d)).

### 3.2. DCA Attenuates Neuronal Apoptosis after I/R Injury

**Figure 2:**
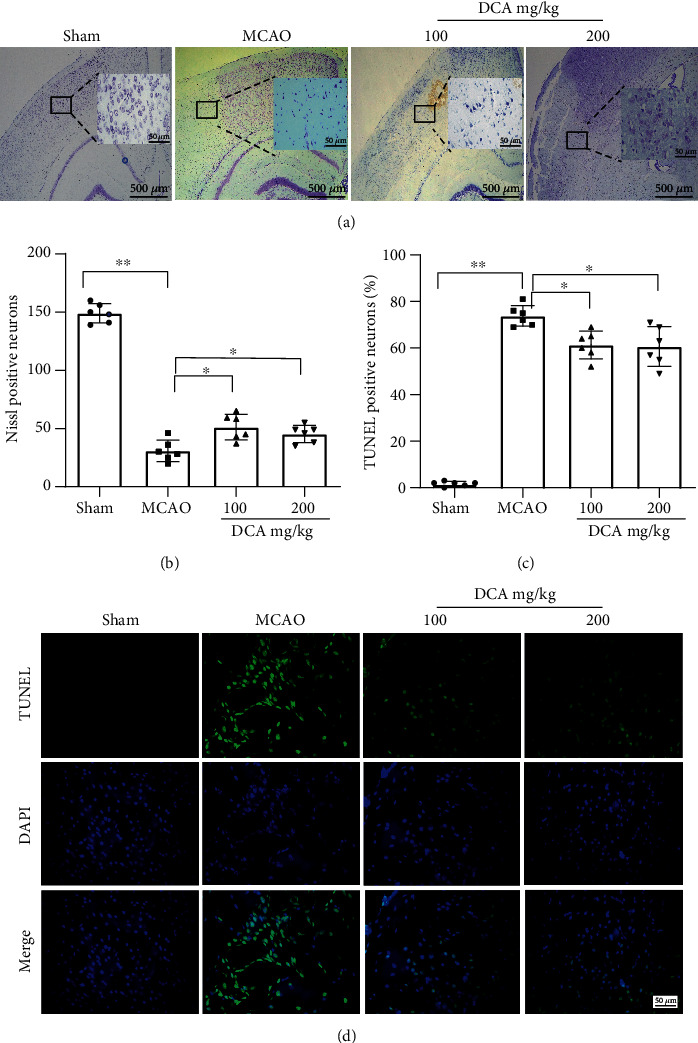
DCA attenuates neuronal apoptosis after brain I/R injury. (a, b) Nissl staining and (c, d) TUNEL staining and quantitative analysis of coronal sections of ischemic cerebral cortex (*n* = 6; scale, 50 *μ*m). ^∗^*P* < 0.05 and ^∗∗^*P* < 0.01. The comparison between cohorts is marked in the figure.

Nissl staining showed a decrease in the number of neurons in the MCAO cohort compared with the sham cohort and a significant improvement in the number of neurons in the DCA treatment cohort (Figures 2(a) and 2(b)). TUNEL staining showed that neuronal apoptosis increased in the MCAO cohort compared with the sham operation cohort but decreased in the DCA cohort (Figures 2(c) and 2(d)).

### 3.3. DCA Attenuates BBB Damage after Cerebral I/R Injury

**Figure 3:**
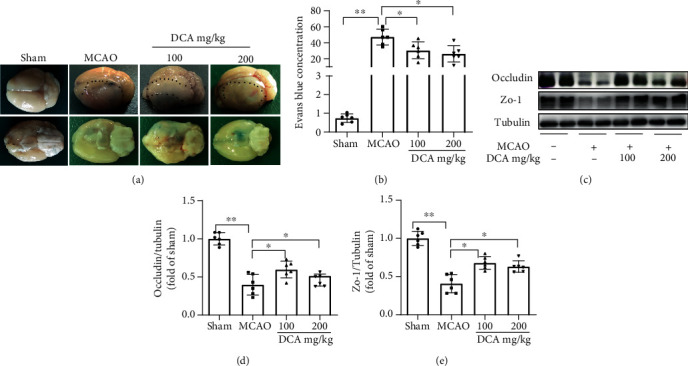
DCA can reduce the damage of the blood-brain barrier attributed to ischemia. (a, b) Representative photograph of Evans blue-stained mouse brains 24 h after sham surgery or MCAO (*n* = 6). (c–e) Western blotting assay of the representative proteins of TJ proteins occludin and ZO-1 in mouse brain and the band strength of the respective protein relative to Tubulin. ^∗^*P* < 0.05 and ^∗∗^*P* < 0.01. The comparison between cohorts is marked in the figure.

The permeability of Evans blue dye is shown in Figures 3(a) and 3(b). Compared with the sham operation cohort, the permeability of Evans blue dye increased in the MCAO cohort but decreased in the DCA cohort. Compared with the sham operation cohort, the MCAO cohort also significantly reduced the expressions of major TJ membrane proteins occludin and ZO-1 (Figures 3(c)–3(e)), which were improved in the DCA treatment cohort, and they interacted to maintain BBB integrity. The mentioned results suggest that DCA inhibits ischemia-induced BBB destruction.

### 3.4. DCA Improves Mitochondrial Metabolism after Cerebral I/R Injury

**Figure 4:**
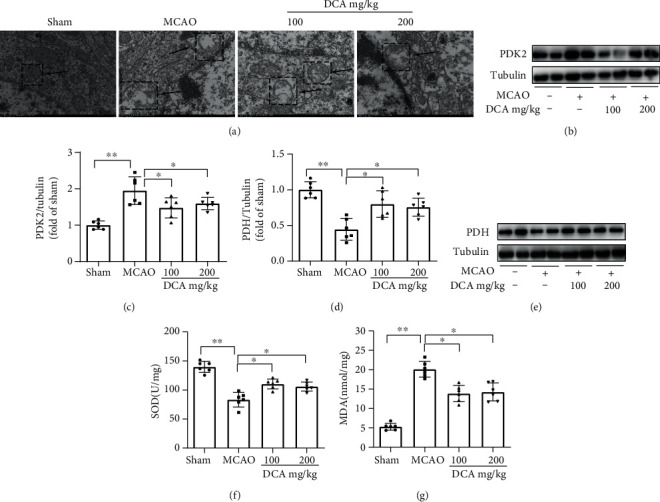
DCA improves mitochondrial metabolism after brain I/R injury. (a) Mitochondrial morphology was observed by electron microscopy 24 h after sham surgery or MCAO (*n* = 6). (b–e) Western blotting assay of key metabolic proteins PDK2 and PDH of mitochondrial TCA cycle in mouse brain as well as the band strength of the respective protein relative to Tubulin. (f, g) Effects of DCA on the contents of oxidative stress products SOD and MDA after brain I/R injury. ^∗^*P* < 0.05 and ^∗∗^*P* < 0.01. The comparison between cohorts has been shown in the figure.

According to electron microscopy (Figure 4(a)), compared with the sham operation cohort, the volume of mitochondria in the MCAO cohort increased, the electron density of matrix decreased, the matrix particles decreased or disappeared, and the cristae became shorter, reduced, and moved to the edge, which was improved in the DCA treatment cohort. In contrast to the sham-operated cohort (Figures 4(b)–4(e)), the expression of mitochondrial metabolism-related protein PDK2 increased, and PDH decreased in the MCAO cohort, PDK2 in the DCA cohort was lower than that in the MCAO cohort, and PDH in the MCAO cohort was higher than that in the MCAO cohort. Oxidative damage was assessed by measuring SOD and MDA productions. As shown in Figures 4(f) and 4(g), as opposed to the sham operation cohort, SOD activity significantly decreased, and MDA content increased in the H/R cohort. Furthermore, DCA administration significantly restored the activity of SOD and decreased the content of MDA after H/R.

### 3.5. DCA Activates the Nrf2/HO-1 Signaling Channel to Reduce Oxidative Damage

**Figure 5:**
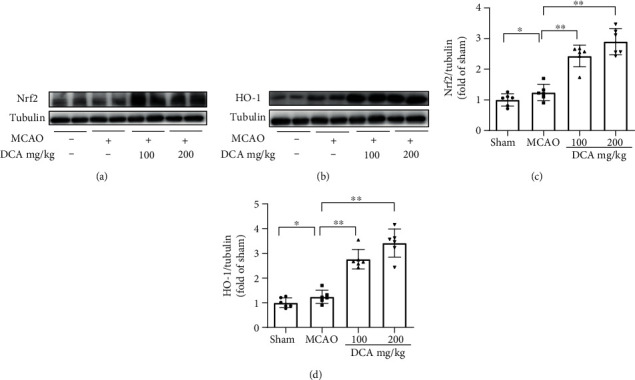
DCA activates the Nrf2/HO-1 signaling channel. (a–d) Western blotting assay of Nrf2 and HO-1 in mouse brains and the band strength of the respective protein relative to Tubulin. ^∗^*P* < 0.05 and ^∗∗^*P* < 0.01. The comparison between cohorts is marked in the figure.

The Western blotting assay was conducted to examine the expressions of oxidative stress-related proteins Nrf2 and HO-1 in the ischemic cerebral cortex. According to Western blotting analysis of the ischemic cerebral cortex after MCAO, DCA significantly upregulated Nrf2 and HO-1 expressions (Figures 5(a)–5(d)).

### 3.6. DCA Improves Mitochondrial Metabolism after I/R Injury and Activates Nrf2/HO-1 Signaling Channel to Reduce Oxidative Damage *In Vitro*

**Figure 6:**
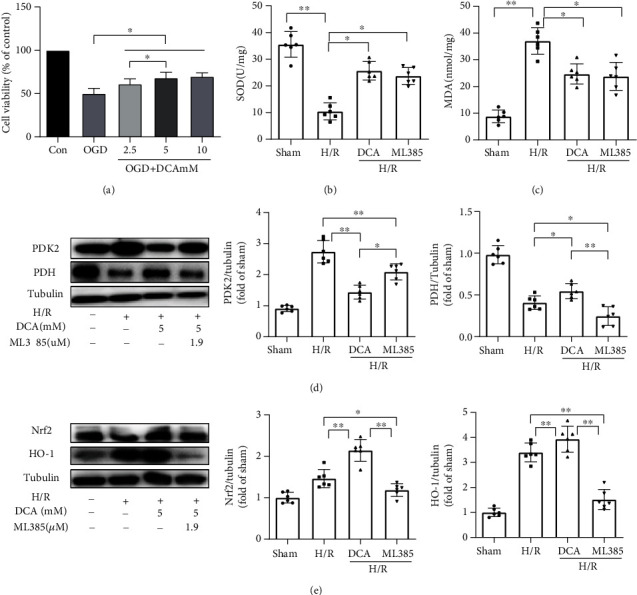
Neuroprotective effect of DCA on HBMEC cells. (a) CCK8 assay was used to detect the viability of cells after injury. (b, c) The productions of SOD and MAD were measured to assess oxidative damage. (d) DCA inhibits PDK2, a key metabolic protein of mitochondrial TCA cycle, and activates the expression of PDH. (e) DCA activated the protein expressions of Nrf2 and HO-1. ^∗^*P* < 0.05 and ^∗∗^*P* < 0.01. The comparison between cohorts is marked in the figure.

The researchers specifically investigated the neuroprotective influence exerted by DCA in HBMEC cells using the OGD model. The viability of the injured cells was measured by the CCK8 assay. For instance (Figure 6(a)), cell viability was significantly reduced by OGD-induced treatment compared with the controls, while cell viability was significantly increased by the DCA treatment. As indicated from the results, DCA could protect differentiated HBMEC cells from OGD injury, and the relative optimal dose was 5 mM.

Oxidative damages were assessed through the measurement of in vitro SOD, MDA, and ROS productions. As shown in Figures 6(b) and 6(c), compared with the sham operation cohort, the SOD activity of the H/R cohort significantly decreased, and the MDA content increased. In addition, DCA significantly restored the activity of SOD and reduced the MDA content after H/R (Supplementary material online, Figure S). DCA reduced the ROS content after H/R. DCA inhibited PDK2, i.e., the key metabolic protein of mitochondrial TCA cycle, and activated PDH. Furthermore, DCA activated Nrf2 and HO-1 expressions (Figures 6(d) and 6(e)). Oxidative damages were assessed through the measurement of SOD, MDA, and ROS productions in vitro.

### 3.7. DCA Attenuates the Damage of Blood-Brain Barrier after I/R Injury *In Vitro* and the Disruption of Tight Protein

**Figure 7:**
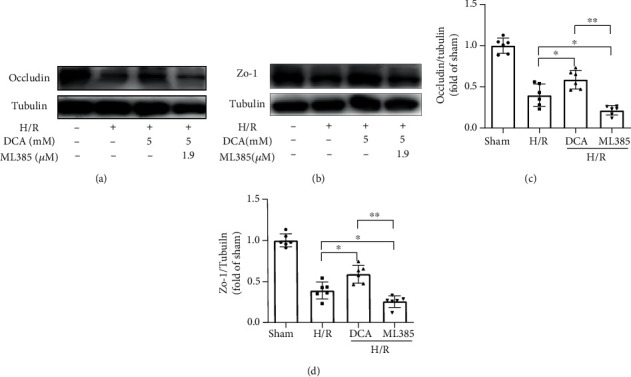
DCA attenuates the expressions of key occludin and ZO-1 of TJ membrane proteins in vitro to maintain BBB integrity. (a–d) Western blotting assay of occludin and ZO-1 and the band strength of the respective protein relative to Tubulin in mouse brain. The mentioned results indicate that DCA inhibits ischemia-induced BBB destruction. ^∗^*P* < 0.05 and ^∗∗^*P* < 0.01. The comparison between cohorts is marked in the figure.

DCA attenuates the expressions of occludin and ZO-1, the key of TJ membrane proteins, in vitro to maintain BBB integrity. The mentioned results indicated that DCA inhibits ischemia-induced BBB destruction (Figures 7(a)–7(d)).

## 4. Discussion

Previous studies have shown that DCA plays an important role in vascular protection [26], promoting vascular revascularization and improving vascular calcification in patients with atherosclerosis [27]. However, the mechanism of DCA regulating mitochondrial metabolism and oxidative stress in cerebral IS and I/R has not been clarified. For IS and I/R, energy metabolism disorder and mitochondrial dysfunction are able to result in considerable free radical formations, thereby triggering oxidative damages, inhibiting the activity of antioxidant enzymes, breaking down the blood-brain barrier, and aggravating brain injury. This study reveals the protective role of DCA in mediating brain I/R injury. DCA can improve CIRI by reducing infarct volume, neurological score, and cerebral water content. DCA administration attenuated mitochondrial metabolism, oxidative stress, neuronal apoptosis, and blood-brain barrier permeability after I/R injury in mice. In addition, the DCA treatment also reduced the mitochondrial metabolism, blood-brain barrier permeability, and oxidative stress in OGD-induced HBMEC cells. This study confirmed that the brain protective function of DCA was related to the activation of the PDK2-PDH-Nrf2 pathway, and the Nrf2-mediated antioxidant stress and blood-brain barrier protection disappeared when ML385, i.e., the specific inhibitor of Nrf2, was used.

Under physiological conditions, ATP required by the brain is mainly produced by pyruvate oxidation (PO) and glucose oxidation (GO) within mitochondria [28]. Pyruvate formation increases the rate of glycolysis and promotes the glucose oxidizing process via the PDH activation [29], thus converting pyruvate to acetyl-coA. Nevertheless, based on pathophysiological conditions (e.g., I/R), due to mitochondrial dysfunction, it can facilitate the expressions of PDK and phosphorylate PDH, thereby inhibiting PDH regulated glucose metabolism and reducing glucose oxidation rate [30, 31]. Studies have found that in the brain, PDK activity mainly has a correspondence to isoenzyme PDK2. Furthermore, DCA could inhibit PDK, the inhibition order was PDK2 > PDK1 > PDK4 > PDK3 [15], which improved the activity of PDH.

Previous studies have shown that DCA is a pharmacological agent that activates PDH by inhibiting PDK and also shows significant neuroprotective potential. The administration of DCA has been suggested to facilitate local lactic acid removal [32], tumor therapy [33], and pulmonary hypertension [34]. However, the protective effect, molecular mechanism, and blood-brain barrier permeability of DCA in cerebral IS and I/R injury have been rarely investigated. This study reported that DCA could exert a protective effect by inhibiting PDK2 and activating PDH to regulate mitochondrial metabolism. The transmission electron microscope was adopted to observe the mitochondria of the cerebral cortex after brain I/R injury, and the results suggested that the mitochondrial injury was alleviated. In IS, after the recovery of cerebral blood flow, considerable free radicals and reactive oxygen species will be produced, leading to the aggravation of I/R injury. The mechanism is that IS leads to the abnormal activities of some enzymes of mitochondrial metabolism (PDK2 and PDH). However, DCA can inhibit PDK2 and activate PDH, thus improving mitochondrial metabolism.

Oxidative stress acts as the vital actor of brain I/R injury, capable of causing neuronal damage and death [35]. Various antioxidants can improve brain I/R injury [36–38]. According to existing studies, DCA exhibits broad biological activity and can cross the blood-brain barrier in mice [39]. Accordingly, we investigated whether DCA affects oxidative stress after brain I/R injury. In addition, Nrf2 refers to a critical antioxidant defense mechanism [40, 41]. Under cerebral I/R, excessive oxidative stress facilitates Keap1 and Nrf2 separating processes, thereby activating Nrf2, and the activated Nrf2 is translocated to the nucleus and binds with ARE, thus activating the transcription of several downstream antioxidant genes [42, 43]. Thus, Nrf2 acts as the vital transcription element for maintaining redox homeostasis. According to existing researches, Nrf2 has a cytoprotective effect within a wide range of I/R-induced brain and kidney injury models [44, 45]. However, there are few studies on how DCA and Nrf2 regulate oxidative stress. We assumed that DCA regulates mitochondrial metabolism by inhibiting PDK2 and activating PDH, thus activating the Nrf2-HO-1 channel to produce antioxidant stress effect. As revealed from the results, the activities of Nrf2 and HO-1 significantly increased after the DCA treatment. We used ML385, a specific inhibitor of Nrf2 in cells, and the protective effect of DCA disappeared, indicating that DCA could alleviate oxidative stress through the PDK2-PDH-Nrf2 channel.

In this study, SOD and MDA were employed to assess oxidative damage. SOD can catalyze superoxide anion free radicals to be transformed into hydrogen peroxide. As a product of lipid peroxidation, MDA has been adopted to assess the level of free radicals within brain I/R injury [46]. Consistent with existing studies, SOD activity declined significantly and the MDA level rose significantly after brain I/R injury. Next, DCA significantly improved the activity of SOD and downregulated MDA levels within brain tissues and HBMEC cells of I/R mice. The mentioned results directly reveal that DCA attenuates brain I/R damage by inhibiting oxidative stress.

The blood-brain barrier (BBB) refers to a selection-related osmotic membrane comprising endothelial cells, extracellular matrix unit pertaining to the basement membrane, pericyte, and endings of astrocytes. The tight junction of endothelial cells is the gatekeeper, restricting the entry of substances from the blood into the brain, thereby maintaining brain homeostasis [47]. After the aggravation of I/R injury, impaired BBB integrity increases paracellular permeability, which allows toxins, a wide range of immune cells, and inflammation-related cytokines to enter the brain, which leads to risen cerebrovascular edema, hemorrhagic transformation, and increased mortality [25]. As indicated from the data of this study, DCA reduces extravasation of Evans blue dye in the I/R cerebral cortex of mice. Moreover, ischemia noticeably reduced the expressions of major TJ membrane protein, including occludin and ZO-1, and DCA significantly improved the expressions of occludin and ZO-1 for maintaining BBB integrity.

The present study also had some defects; for example, BBB only observed changes in endothelial cells but did not observe the relationship between the extracellular matrix components of the basement membrane, pericytes and astrocytes, and BBB. In addition, we did not observe the relevant studies on DCA in patients, and the relevant mechanisms need to be further studied.

**Figure 8:**
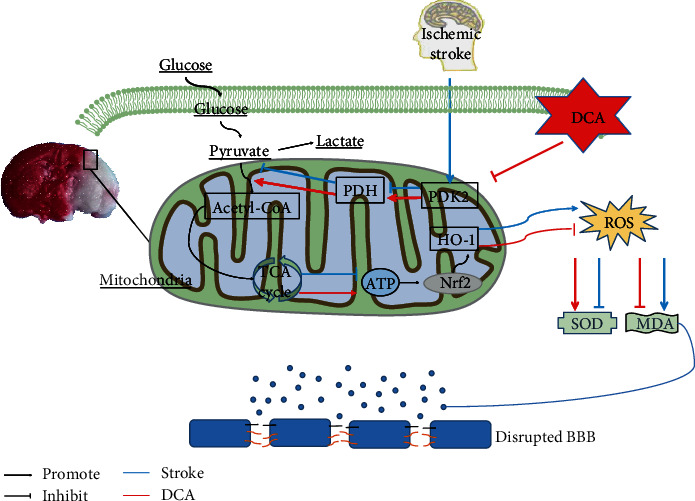
Sodium dichloroacetate (DCA) improves glycolysis by inhibiting PDK2 and activating PDH and promotes the conversion of pyruvate into acetyl-CoA, which then enters the TCA cycle so as to produce ATP, thus improving glycolysis. Meanwhile, it activates the Nrf2/HO-1 signaling pathway to increase the activity of SOD, inhibit the generation of MDA, and reduce oxidative stress, thereby alleviating the damage of the blood-brain barrier and facilitating the recovery of oxidative metabolism.

In brief, our study proved that after brain I/R injury, DCA can improve the activity of mitochondrial-related enzymes PDK2/PDH to promote energy generation and activate the Nrf2 pathway to inhibit oxidative stress and neuronal apoptosis and increase BBB permeability (Figure 8). The PDK2-PDH-Nrf2 pathway may play a key role and provide a new pharmacology target in cerebral IS and I/R protection by DCA.

## Data Availability

The data used to support the findings of this study are available from the corresponding authors upon request.

## Supplementary Materials

Supplementary Materials Supplementary Figure: effect of DCA on ROS levels in HBMEC cells induced by H/R. (a) Reactive oxygen species levels were analyzed by DCFH-DA kit. (b) Compared with the sham operation cohort, the ROS content increased. In addition, DCA reduced the ROS content after H/R. ^∗^*P* < 0.05 and ^∗∗^*P* < 0.01. The comparison between cohorts is marked in the figure.
