# Supplementary figures and images for: DCA Protects against Oxidation Injury Attributed to Cerebral Ischemia-Reperfusion by Regulating Glycolysis through PDK2-PDH-Nrf2 Axis
Source: Oxid Med Cell Longev. 2021 Oct 19;2021:5173035. doi: 10.1155/2021/5173035 (PMC8548159; doi:10.1155/2021/5173035)

ROS

Sham

H/R

DCA

ML385

H/R+DCA mM

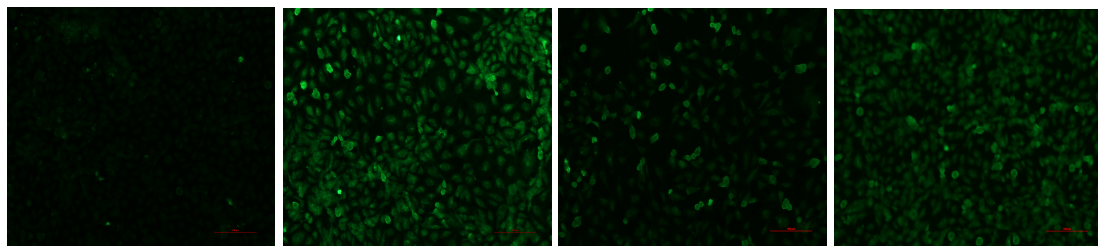

(a)

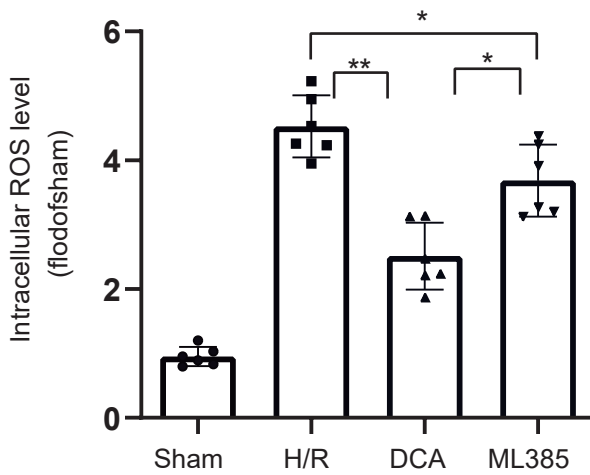

(b)

H/R

Supplement: Supplementary Materials — Supplementary Figure: effect of DCA on ROS levels in HBMEC cells induced by H/R. (a) Reactive oxygen species levels were analyzed by DCFH-DA kit. (b) Compared with the sham operation cohort, the ROS content increased. In addition, DCA reduced the ROS content after H/R. ∗P < 0.05 and ∗∗P < 0.01. The comparison between cohorts is marked in the figure. [file 5173035.f1.pdf]
